# A mentor-mentee support program for people with anorexia nervosa

**DOI:** 10.1186/2050-2974-3-S1-O45

**Published:** 2015-11-23

**Authors:** Lucie Ramjan, Sarah Fogarty, Daniel Nicholls, Phillipa Hay

**Affiliations:** 1 School of Nursing and Midwifery; University of Western Sydney, Australia; 2 School of Medicine; University of Western Sydney, Australia

## Background

Successful mentorship enhances quality of life, hope for recovery and empowers people with anorexia nervosa (AN) to develop health-promoting strategies, with immeasurable benefits for both mentor and mentee.

## Aims

To develop and evaluate, in consultation with stakeholders (people with AN and recovered individuals), a mentorship support program for people with AN in NSW.

## Methods

This study is a mixed methods participatory action research (PAR) project. Participants are pivotal in the development and design of a tailored program. Researchers work closely with stakeholders to bring and evaluate change, through cycles of learning and reflection, actively engaging participants in the research process.

## Results

During workshop discussions, participants (4 mentors and 5 mentees) defined the program, examined roles and responsibilities, partnerships, interaction, use of funds, conflict resolution and risk management. The workshop, conducted informally in a setting that simulated a home environment, reduced anxiety levels. Uncertainty evolved into an appreciation for the program, development of new relationships, learning that everyone had similar reservations yet reassurance that people were there to support and encourage recovery. Participants were positive and optimistic for the program's trial.

## Conclusion

PAR is an apt research method for actively engaging stakeholders in the development of a tailored program to support recovery.

